# Supplementary material for: Resolving the full spectrum of human genome variation using Linked-Reads
Source: Genome Res. 2019 Apr;29(4):635–45. doi: 10.1101/gr.234443.118 (PMC6442396; doi:10.1101/gr.234443.118)
Supplement: Supplemental Material [file supp_gr.234443.118_Supplemental_longranger-2.2.zip › longranger-2.2.tar.bz2!/lr2.2-public/lib/stan/cmdstan/stan/doc/api/html/classstan_1_1agrad_1_1_l_d_l_t__alloc.html]

Stan: stan::agrad::LDLT\_alloc< R, C > Class Template Reference


|  |  |
| --- | --- |
|  | Stan  2.5.0  probability, sampling & optimization |


- Main Page
- Related Pages
- Namespaces
- Classes
- Files

- Class List
- Class Index
- Class Hierarchy
- Class Members

All Classes Namespaces Files Functions Variables Typedefs Enumerations Enumerator Friends Macros Pages

- stan
- agrad
- LDLT\_alloc

Public Member Functions |
Public Attributes |
List of all members

stan::agrad::LDLT\_alloc< R, C > Class Template Reference

This object stores the actual (double typed) LDLT factorization of an Eigen::Matrix<var> along with pointers to its vari's which allow the \*\_ldlt functions to save memory.
More...

`#include <LDLT_alloc.hpp>`

Inheritance diagram for stan::agrad::LDLT\_alloc< R, C >:

|  |  |
| --- | --- |
| Public Member Functions | |
|  | LDLT\_alloc () |
|  | |
|  | LDLT\_alloc (const Eigen::Matrix< var, R, C > &A) |
|  | |
| void | compute (const Eigen::Matrix< var, R, C > &A) |
|  | Compute the LDLT factorization and store pointers to the vari's of the matrix entries to be used when chain() is called elsewhere. More... |
|  | |
| double | log\_abs\_det () const |
|  | Compute the log(abs(det(A))). This is just a convenience function. More... |
|  | |
| Public Member Functions inherited from stan::agrad::chainable\_alloc | |
|  | chainable\_alloc () |
|  | |
| virtual | ~chainable\_alloc () |
|  | |

|  |  |
| --- | --- |
| Public Attributes | |
| size\_t | N\_ |
|  | |
| Eigen::LDLT< Eigen::Matrix  < double, R, C > > | \_ldlt |
|  | |
| Eigen::Matrix< vari \*, R, C > | \_variA |
|  | |

## Detailed Description

### template<int R, int C> class stan::agrad::LDLT\_alloc< R, C >

This object stores the actual (double typed) LDLT factorization of an Eigen::Matrix<var> along with pointers to its vari's which allow the \*\_ldlt functions to save memory.

It is derived from a chainable\_alloc object so that it is allocated on the stack but does not have a chain() function called.

This class should only be instantiated as part of an LDLT\_factor object and is only used in \*\_ldlt functions.

Definition at line 20 of file LDLT\_alloc.hpp.

## Constructor & Destructor Documentation

template<int R, int C>

|  |  |  |  |  |
| --- | --- | --- | --- | --- |
| stan::agrad::LDLT\_alloc< R, C >::LDLT\_alloc | ( |  | ) |  |

inline

Definition at line 22 of file LDLT\_alloc.hpp.

template<int R, int C>

|  |  |  |  |  |  |
| --- | --- | --- | --- | --- | --- |
| stan::agrad::LDLT\_alloc< R, C >::LDLT\_alloc | ( | const Eigen::Matrix< var, R, C > & | *A* | ) |  |

inline

Definition at line 23 of file LDLT\_alloc.hpp.

## Member Function Documentation

template<int R, int C>

|  |  |  |  |  |  |
| --- | --- | --- | --- | --- | --- |
| void stan::agrad::LDLT\_alloc< R, C >::compute | ( | const Eigen::Matrix< var, R, C > & | *A* | ) |  |

inline

Compute the LDLT factorization and store pointers to the vari's of the matrix entries to be used when chain() is called elsewhere.

Definition at line 32 of file LDLT\_alloc.hpp.

template<int R, int C>

|  |  |  |  |  |
| --- | --- | --- | --- | --- |
| double stan::agrad::LDLT\_alloc< R, C >::log\_abs\_det | ( |  | ) | const |

inline

Compute the log(abs(det(A))). This is just a convenience function.

Definition at line 49 of file LDLT\_alloc.hpp.

## Member Data Documentation

template<int R, int C>

Eigen::LDLT<Eigen::Matrix<double,R,C> > stan::agrad::LDLT\_alloc< R, C >::\_ldlt

Definition at line 54 of file LDLT\_alloc.hpp.

template<int R, int C>

Eigen::Matrix<vari\*,R,C> stan::agrad::LDLT\_alloc< R, C >::\_variA

Definition at line 55 of file LDLT\_alloc.hpp.

template<int R, int C>

size\_t stan::agrad::LDLT\_alloc< R, C >::N\_

Definition at line 53 of file LDLT\_alloc.hpp.

---

The documentation for this class was generated from the following file:

- src/stan/agrad/rev/matrix/LDLT\_alloc.hpp


---

[ Stan Home Page ]

*© 2011–2014,
Stan Development Team.*
